# Supplementary material for: Carbohydrates and the oxidative branch of the pentose phosphate pathway modify Bacteroides thetaiotaomicron phage resistance by phase-variable S-layers
Source: J Bacteriol. 2025 Sep 12;207(10):e00178-25. doi: 10.1128/jb.00178-25 (PMC12548429; doi:10.1128/jb.00178-25)
Supplement: Supplemental figures — Fig. S1 to S5. [file jb.00178-25-s0001.docx]

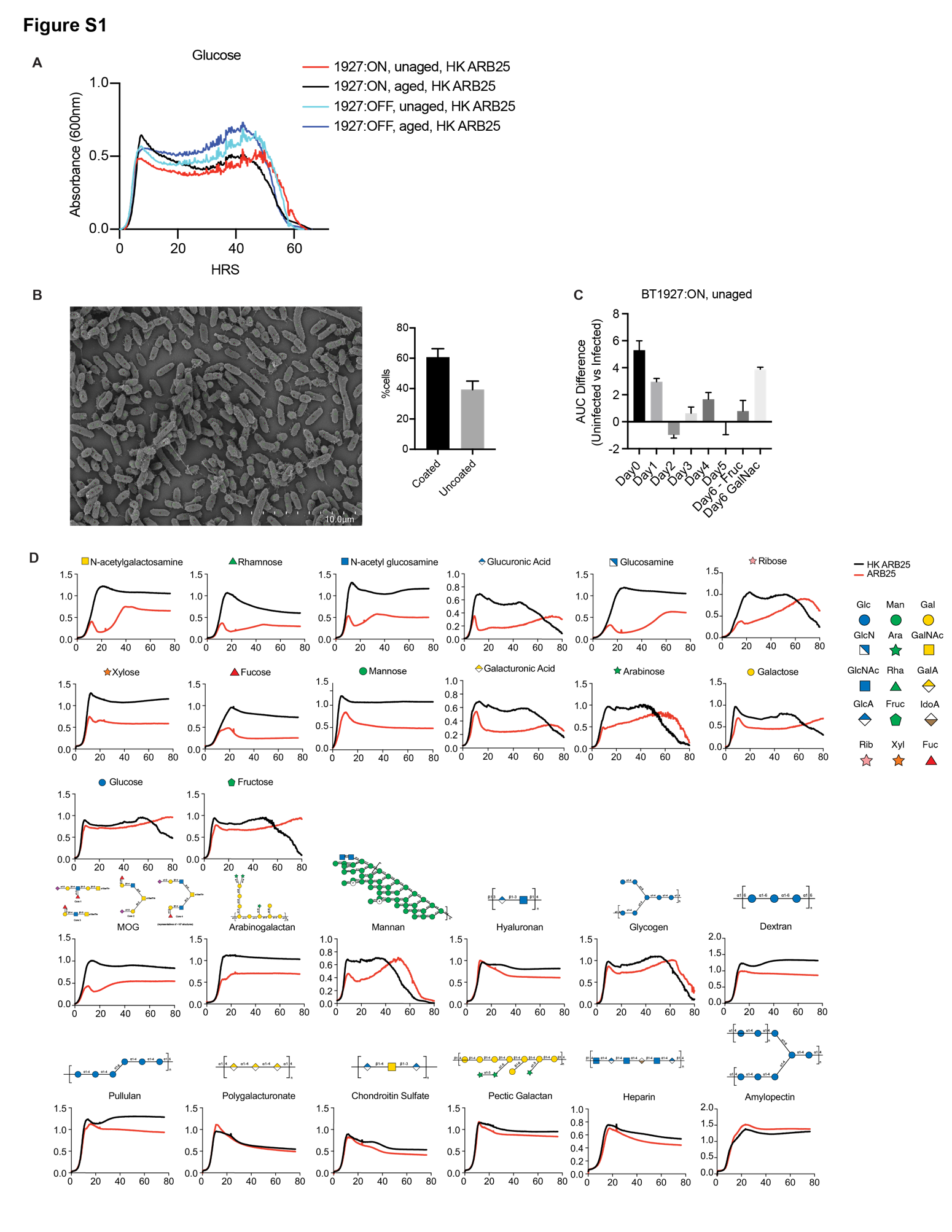


**Figure S1.** **Effects of colony aging and different carbohydrates on BT1927-mediated phage resistance.** (**A**) Growth curves for BT1927:ON or BT1927:OFF unaged or aged strains treated with HK ARB25, which are additional controls for **Figure 1A**. (**B**) SEM imaging and quantification of the BT1927:ON strain showing cells that appear to exhibit the BT1927-associated crystalline “coating” over most of their surface (pink dot, green circle, black bar) and what appear to be “uncoated” cells (green circle only, grey bar) The plot at left shows the results of 3 unblinded counts by two different experimenters. (**C**) Area under the curve (AUC) differences within the first 24-hour of growth for unaged (*i.e*., grown directly from freezer) BT1927:ON strain grown successively for 5-days of passage in glucose and subsequent passage
(day 6) into fructose or GalNAc. (**D**) Growth of the BT1927:ON strain in BPRM medium containing the different carbohydrates noted as the sole carbon source along with their corresponding glycan symbol above each graph (5mg/ml, n= 3 per condition).

**
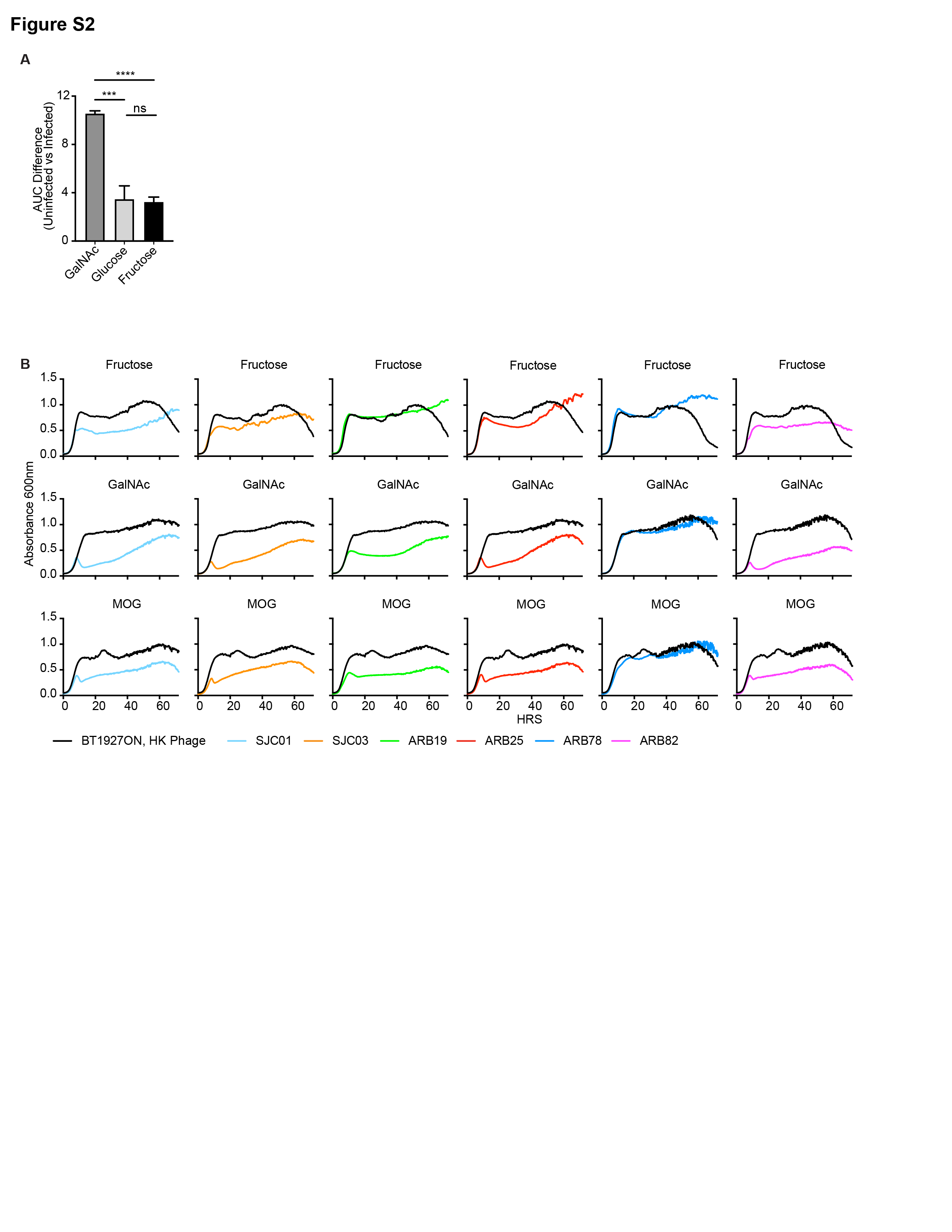
**

**Figure S2.** **Growth on N-acetylgalactosamine increases susceptibility to multiple phage**. (**A**) AUC differences within the first 24-hour of growth­­ between ARB25 infected and HK control for cultures grown in GalNAc, glucose, or fructose, corresponding to **Figure 3B,C** (Two-tailed t-test, ***P=0.0009, ****P<0.0001). (**B**) Growth curves of the BT1927:ON strain treated with HK *B. thetaiotaomicron* phage (black) or challenged with the 6 indicated *B. thetaiotaomicron* phages in BPRM containing fructose (top) GalNAc (middle) or mucin *O*-glycans (MOG; Bottom), n = 3 per condition.

**
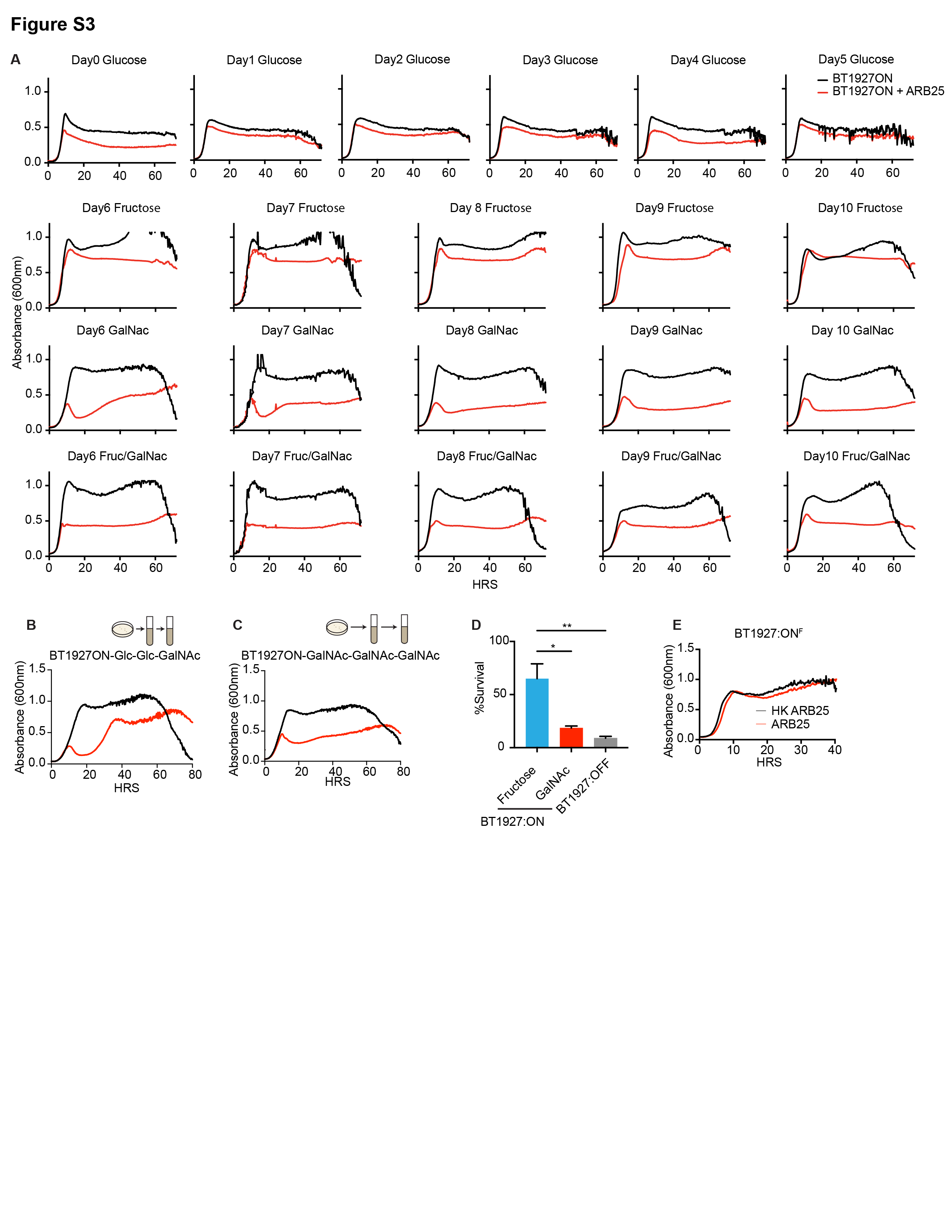
**

**Figure S3.** **N-acetylgalactosamine elicits higher ARB25 susceptibility in the presence of fructose and increases complement killing.** (**A**) Growth of successively passaged BT1927:ON cultures in BPRM medium containing glucose for 5 days and subsequent growth after passage (day 6 and later) into fructose, GalNAc, or a 1:1 mixture of fructose and GalNAc (at 5mg/ml each) as indicated for days 6-10, n = 3 per condition. (**B**) ARB25 infection kinetics for the BT1927:ON strain grown on solid BPRM medium containing glucose, grown in liquid culture containing glucose and sub-cultured again into liquid media containing GalNAc (as indicated above plot) and treated with HK ARB25 (black) or ARB25 phage (red). (**C**) ARB25 infection kinetics for the BT1927:ON strain grown on a solid BPRM medium containing GalNAc, grown in liquid culture containing GalNAc and sub-cultured again into liquid media containing GalNAc (as indicated above plot) treated with HK ARB25 (black) or ARB25 phage (red). (**D**) Percent survival of BT1927:ON grown in either fructose (blue) or GalNAc (red) or the BT1927:OFF strain grown in fructose (grey) challenged with pooled human complement and normalized to a heat-denatured complement control. (**E**) Growth of the epitope-tagged BT1927:ON^F^ strain in the presence of HKARB25 (black) or ARB25 (red), demonstrating the FLAG tag does not inhibit BT1927-mediated resistance.

**
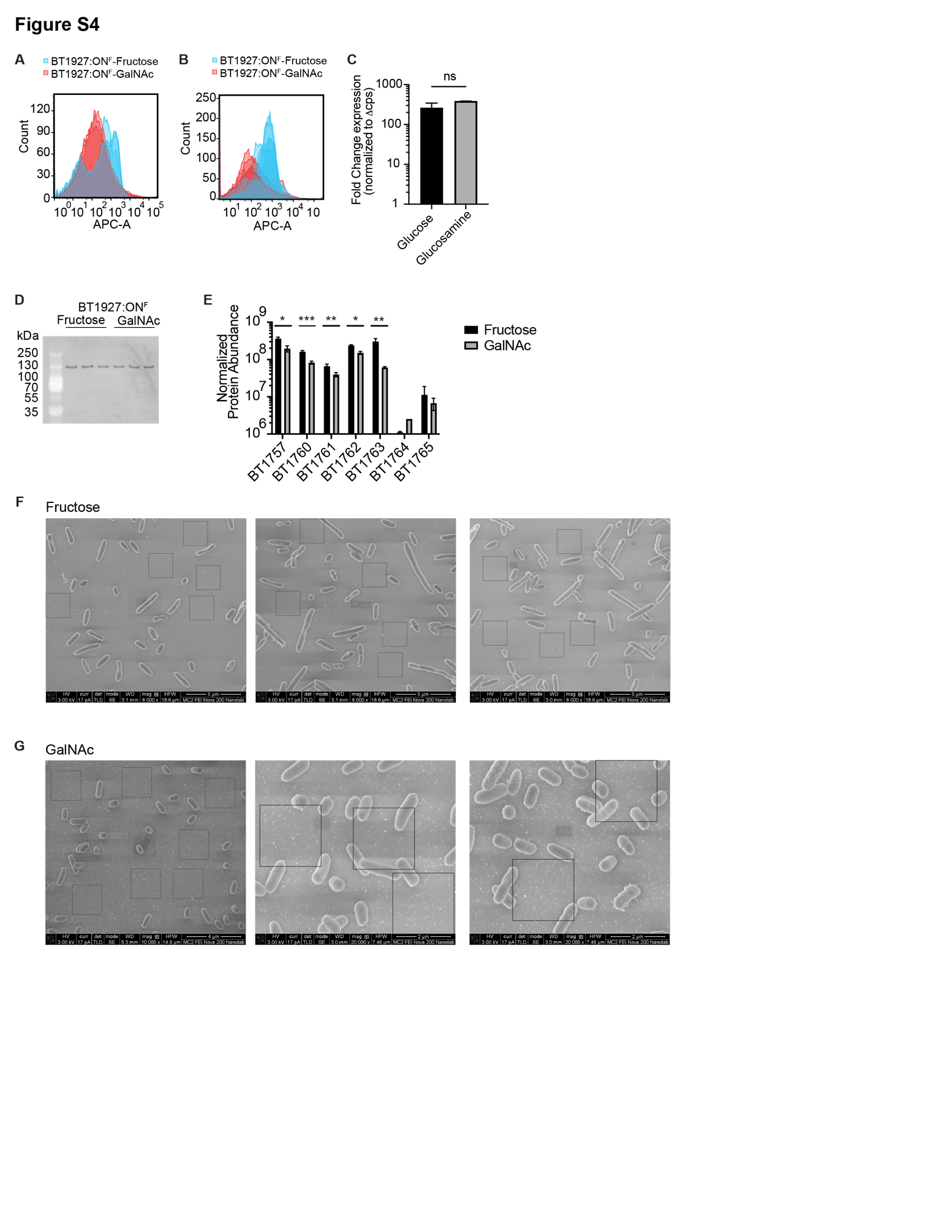
**

**Figure S4. Sugars that increase ARB25 susceptibility do not decrease BT1927 transcript or cell abundance but do decrease surface display and increase vesiculation.** (**A**) Flow cytometry histograms for the BT1927:ON^F^ strain grown in BPRM medium containing fructose or GalNAc from an experiment in which all 3 fructose replicates showed a BT1927 low staining population. (**B**) Flow cytometry histogram for the BT1927:ON^F^ strain grown in BPRM medium containing fructose or GalNAc from an experiment in which replicates were grown in different anaerobic chamber and only one replicate showed a BT1927 low staining population. (**C**) *BT1927* transcript expression in the BT1927:ON strain grown in glucose (black) or glucosamine (grey), another sugar that results in increased ARB25 susceptibility, at mid-log phase normalized to the acapsular (Δcps) control. (**D**) Western blot of whole cells of the BT1927:ON^F^ strain grown in either fructose or GalNAc and probed with α-FLAG antibody. (**E**) Normalized protein abundance for products of the levan associated PUL that is known to be induced by fructose (two-tailed t-test, P *<0.001, **<0.05, *** <0.005). (**F**) SEM images of the BT1927:ON strain grown in fructose from which OMV measurements were made. (**G**) SEM images of the BT1927:ON strain grown in GalNAc from which OMV measurements were made. In F and G, black boxes indicate normalized area used for blinded counting of OMV particles (note that images vary in magnification and boxes are scaled accordingly).

**
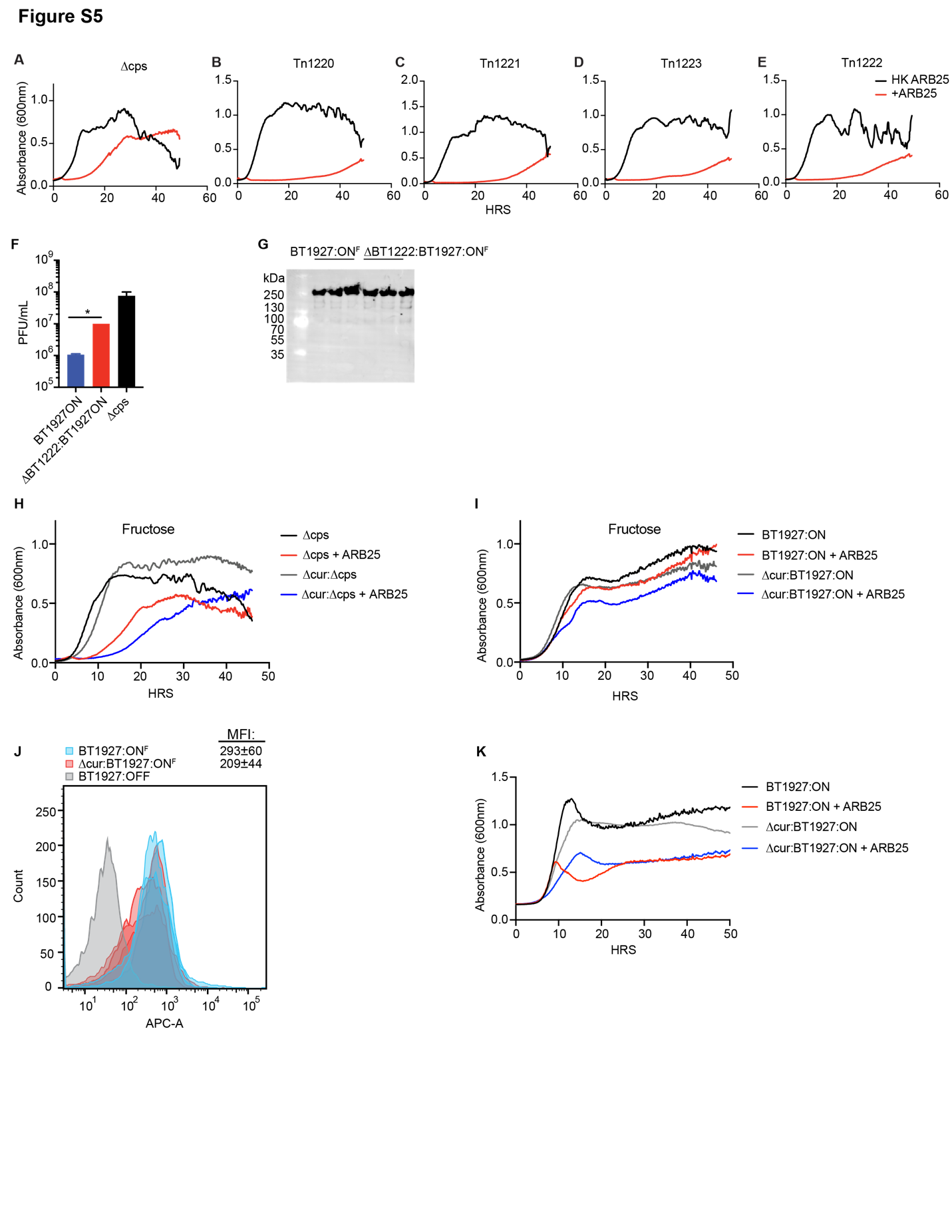
**

**Figure S5.** **Mutants in the oxidative PPP and an associated carboxylate transporter, but not Cur, increase ARB25 susceptibility in glucose.** (**A-E**) Growth of the acapsular *B. thetaiotaomicron* strain (Δcps) and transposon mutants in the Δcps strain background with insertions in genes encoding the 3 steps of the oxidative branch of the pentose phosphate pathway and a linked genes encoding a putative carboxylate transporter (BT1223) grown in BPRM fructose. In each panel, cultures were treated with HK ARB25 (black) or ARB25 (red). (**F**) Quantification of plaques formed on the BT1927:ON strain (blue), a *BT1222* deletion in the 1927:ON strain background (red) or the more susceptible Δcps strain (black). (**G**) Western blot using α-FLAG antibody to detect BT1927 in the BT1927:ON^F^ strain or the isogenic Δ*BT1222* mutant grown in BPRM fructose. (**H**) Growth of the Δcps or Δcps,Δ*cur* deletion mutants in the presence of HK ARB25 or ARB25 (red or blue, respectively). (**I**) Growth of the BT1927:ON (black) or corresponding Δ*cur* deletion mutant (grey) in the presence of HK ARB25 or ARB25 (red or blue, respectively). (**J**) Flow cytometry histogram staining for the epitope-tagged BT1927 (APC-A) in either the BT1927:ON^F^ strain (blue) or a Δ*cur* mutant in this same background (red), grown in fructose. MFI values are shown and statistical comparison reported in the main text. (**K**) Growth curves of the BT1927:ON strain in GlcNAc and treated with HK ARB25 (black) or ARB25 (red) or *cur* mutant in the same BT1927:ON strain background treated with HK ARB25 (grey) or ARB25 (blue).
